# Molecular Mechanisms Involved in Vascular Interactions of the Lyme Disease Pathogen in a Living Host

**DOI:** 10.1371/journal.ppat.1000169

**Published:** 2008-10-03

**Authors:** M. Ursula Norman, Tara J. Moriarty, Ashley R. Dresser, Brandie Millen, Paul Kubes, George Chaconas

**Affiliations:** 1 Department of Physiology & Biophysics, University of Calgary, Calgary, Alberta, Canada; 2 Departments of Biochemistry & Molecular Biology and Microbiology & Infectious Diseases, University of Calgary, Calgary, Alberta, Canada; Medical College of Wisconsin, United States of America

## Abstract

Hematogenous dissemination is important for infection by many bacterial pathogens, but is poorly understood because of the inability to directly observe this process in living hosts at the single cell level. All disseminating pathogens must tether to the host endothelium despite significant shear forces caused by blood flow. However, the molecules that mediate tethering interactions have not been identified for any bacterial pathogen except *E. coli*, which tethers to host cells via a specialized pillus structure that is not found in many pathogens. Furthermore, the mechanisms underlying tethering have never been examined in living hosts. We recently engineered a fluorescent strain of *Borrelia burgdorferi,* the Lyme disease pathogen, and visualized its dissemination from the microvasculature of living mice using intravital microscopy. We found that dissemination was a multistage process that included tethering, dragging, stationary adhesion and extravasation. In the study described here, we used quantitative real-time intravital microscopy to investigate the mechanistic features of the vascular interaction stage of *B. burgdorferi* dissemination. We found that tethering and dragging interactions were mechanistically distinct from stationary adhesion, and constituted the rate-limiting initiation step of microvascular interactions. Surprisingly, initiation was mediated by host Fn and GAGs, and the Fn- and GAG-interacting *B. burgdorferi* protein BBK32. Initiation was also strongly inhibited by the low molecular weight clinical heparin dalteparin. These findings indicate that the initiation of spirochete microvascular interactions is dependent on host ligands known to interact *in vitro* with numerous other bacterial pathogens. This conclusion raises the intriguing possibility that fibronectin and GAG interactions might be a general feature of hematogenous dissemination by other pathogens.

## Author Summary

Many bacterial pathogens can cause systemic illness by disseminating through the blood to distant target sites. However, hematogenous dissemination is still poorly understood, in part because of an inability to directly observe this process in living hosts in real time and at the level of individual pathogens. We recently engineered a fluorescent strain of *Borrelia burgdorferi,* the Lyme disease pathogen, and visualized its dissemination from the microvasculature of living mice using intravital microscopy. We found that dissemination was a multistage process that included tethering, dragging, stationary adhesion and extravasation. In the study described here, we used quantitative real-time intravital microscopy to investigate the mechanistic features of the vascular interaction stage of *B. burgdorferi* dissemination in living hosts. We found that tethering and dragging interactions (collectively referred to as initiation interactions) were mechanistically distinct from stationary adhesion. Initiation of microvascular interactions required the *B. burgdorferi* protein BBK32, and host ligands fibronectin and glycosaminoglycans. Initiation interactions were also strongly inhibited by the low molecular weight clinical heparin dalteparin. Since numerous bacterial pathogens can interact with fibronectin and glycosaminoglycans *in vitro*, these observations raise the intriguing possibility that fibronectin and glycosaminoglycan recruitment might be a feature of hematogenous dissemination by other pathogens.

## Introduction

Hematogenous dissemination of pathogenic organisms is an important feature of disease progression. However, dissemination is poorly understood, in large part because of the difficulty in studying this process directly in living organisms under the shear stress conditions that characterize the host vasculature. One such disseminating pathogen is the spirochete *Borrelia burgdorferi*, a primarily extracellular bacterium causing Lyme disease, also referred to as Lyme borreliosis [1].

Pathogenic spirochetes cause a number of emerging and re-emerging diseases, including syphilis, leptospirosis, relapsing fever and Lyme disease [2]–[5]. *B. burgdorferi* is transmitted to the dermis of vertebrate hosts during the blood meal of *Ixodes* ticks, and subsequently disseminates to other tissues and organs during the hematogenous phase of infection [1]. *B. burgdorferi* and other spirochetes interact with endothelial cells under static conditions *in vitro* [6]–[8]. However, until recently, spirochete-vascular interactions have never been directly examined in the host itself, or under the fluid shear forces that are present at dissemination sites [9].

**Figure 1:**
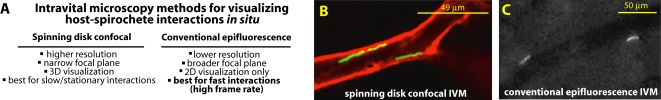
Intravital microscopy to study *B. burgdorferi* microvascular interactions *in vivo.* A) Table summarizing the properties of spinning disk confocal and conventional epifluorescence intravital microscopy (IVM) with respect to visualization of *B. burgdorferi* vascular interactions *in situ*. B) Spinning disk confocal and C) conventional epifluorescence micrographs of fluorescent infectious *B. burgdorferi* in the skin microvasculature of a living mouse. Blood vessels in b) were visualized using AlexaFluor555-conjugated antibody to PECAM-1 (red). Spinning disk confocal and conventional epifluorescence IVM videos from the microvasculature are presented in Videos S1 and S2, respectively.

To facilitate direct study of hematogenous dissemination we recently generated a fluorescent infectious strain of *B. burgdorferi*, and used intravital microscopy (IVM) to directly visualize its interaction with and extravasation from the microvasculature of living murine hosts (as summarized in **Fig. 1A–C**) [9]. IVM is a powerful tool for studying the dissemination and transmigration of tumor and immune cells in living hosts, but it is only recently that this technique has begun to be applied to the study of host-pathogen interactions [10],[11].

The results of our recent study indicated that *B. burgdorferi* dissemination from the host microvasculature *in vivo* is a progressive, multi-stage process consisting of several successive steps: transient and dragging interactions (collectively referred to as short-term interactions), followed by stationary adhesion and extravasation. Short-term interactions constitute the majority of spirochete-endothelial associations (89% and 10% for transient and dragging interactions, respectively), take less than one second (transient interactions) or 3–20s (dragging interactions) to travel 100 µm along the vessel wall, and occur primarily on the surface of endothelial cells and not at endothelial junctions [9]. Transient interactions are characterized by a tethering-type attachment-detachment cycle of association in which part of the spirochete adheres briefly to the endothelium before being displaced by blood flow, whereas dragging spirochetes adhere along much of the length of the bacterium, and creep more slowly along the vessel wall [9]. In contrast, stationary adhesions (1% of interactions) do not move along the vessel wall, occur chiefly, but not exclusively, at endothelial junctions, and entail a more intimate association with the endothelium than short-term interactions [9]. Finally, spirochete extravasation (<0.12% of interactions) also occurs primarily, but not exclusively, at endothelial junctions, and is a triphasic process consisting of a rapid, end-first initial penetration of the endothelium, followed by a prolonged period of reciprocating movement, and ending with a rapid exit phase in which the bacterium bursts out of the vessel and migrates rapidly into the surrounding tissue [9].

*In vitro* studies have shown that *B. burgdorferi* binds several host molecules that might mediate endothelial interactions *in vivo*, including fibronectin (Fn), integrins, heparan sulfate-type glycosaminoglycans (GAGs) and regulators of the complement cascade [12]-[20]. A broad array of pathogens have been shown to interact with these ubiquitous host molecules in direct binding assays and tissue culture models *in vitro*; most of these studies have been performed in the absence of shear forces, microvascular endothelium or a functioning immune system, and so the potential contribution of such interactions to hematogenous dissemination in the living host is unknown. To date, 19 candidate adhesin genes have been identified in *B. burgdorferi*, two of which are known to interact with integrins (P66 and BBB07), and two of which can associate with heparan sulfate GAGs (BBK32 and Bgp) [16], [17], [19], [21]–[25]. *B. burgdorferi* encodes one characterized Fn binding protein, BBK32, and appears to express a number of others [12],[16],[24]. Five *B. burgdorferi* CRASP proteins that interact with complement cascade regulating proteins factor H, FHL-1 and FHR-1 have also been identified [20],[26],[27], but their potential contributions to endothelial cell adhesion are unknown.

In the work described here we used IVM to explore the mechanistic basis for *B. burgdorferi* interactions with the microvasculature of living mice. We found that the initiating and stationary adhesion stages of microvascular interactions were mechanistically distinct but inter-dependent events, and that BBK32, Fn and GAGs played a substantial role in initiation events. These findings and the methodology described here provide a framework for investigating the role of Fn and GAGs in vascular interactions during hematogenous dissemination by *B. burgdorferi* and possibly other pathogens.

## Results

### Host GAGs promote *B. burgdorferi* interactions with the microvasculature *in vivo*

To quantitatively analyze *B. burgdorferi* interactions with the microvascular endothelium *in vivo* we employed conventional epifluorescence IVM to examine interactions in the flank skin of mice after intravenous inoculation with 4×10^8^ spirochetes (**Fig. 1** and **Videos S1** and **S2**). Conventional epifluorescence IVM was used instead of spinning disk confocal IVM because it is more effective for imaging the rapid associations that constitute the bulk of *B. burgdorferi* microvascular interactions (**Fig. 1**). Analysis was performed in post-capillary venules, where interactions could be most accurately quantified. For all experiments reported in this manuscript, data describing the numbers of recorded vessels and mice for each experimental condition, as well as the average time after spirochete injection at which recordings were made are provided in the **Figure Legends**. As we have recently reported, during the experimental observation period no signs of endothelial or leukocyte activation were detected [9]. In addition, leukocyte adhesion in dermal postcapillary venules is an indicator of local activation, and can be measured by using the dye rhodamine 6G to fluorescently label all circulating leukocytes and then counting the number of adherent leukocytes in a 100 µm length of vessel [28]. The presence of infectious *B. burgdorferi* in the vasculature for as long as 70 minutes after injection of spirochetes did not significantly alter leukocyte adhesion from the baseline levels observed in the absence of *B. burgdorferi* (1.56−/+0.29 vs 1.75−/+1.15 adhered leukocytes/100 µm, respectively; P = 0.797; N = 50 vessels from 5 mice). The observed number of leukocyte adhesions was normal for the dermal microvasculature of mice [28].

**Figure 2:**
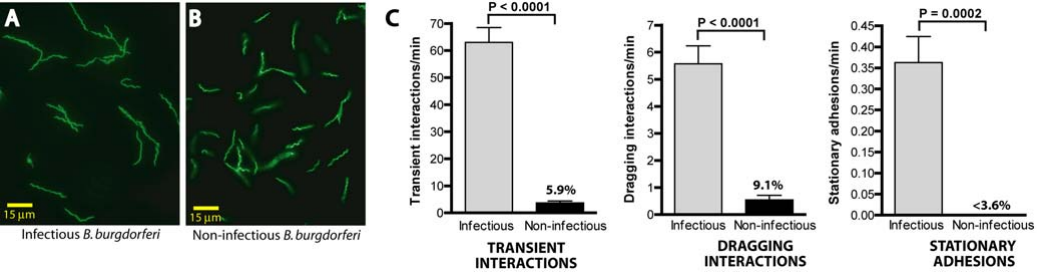
Interaction of infectious and non-infectious fluorescent *B. burgdorferi* with the microvasculature of a living murine host. Micrographs of infectious (A) and non-infectious (B) GFP-expressing *B. burgdorferi* visualized on slides by epifluorescence microscopy. (C) Graphical summary of infectious and non-infectious *B. burgdorferi* interactions in postcapillary venules of the skin microvasculature. The number of interactions/minute in each interaction class was determined by measuring interactions from video footage of conventional epifluorescence IVM. The percentages above the bars for non-infectious spirochetes indicate the non-infectious interaction rate expressed as a percentage of the interaction rate of infectious spirochetes. Sample footage of the videos used to measure spirochete interactions is presented in Video S2. A total of 8,343 spirochete interactions in 85 venules from 17 mice (n = 8 and n = 9, respectively, for experiments performed with non-infectious and infectious *B. burgdorferi*) were analyzed. Standard error bars are indicated for each interaction class. P-values for this figure and all others were determined using a two-tailed non-parametric Student's t-test. Microvascular interactions were measured between 5 and 45 minutes after spirochete injection. The average time after injection for all recordings made with infectious and non-infectious *B. burgdorferi* was 15.9−/+8.1 and 14.1−/+9.3 min, respectively.

As shown in **Fig. 2**, the ability to interact with the microvascular endothelium was specific to infectious spirochetes. When mice were injected with non-infectious *B. burgdorferi* exhibiting the same fluorescence intensity as infectious spirochetes (**Fig. 2A and B**), transient interactions were reduced by 94% (**Fig. 2C**). Furthermore, non-infectious *B. burgdorferi* did not form many dragging interactions and no detectable stationary adhesions (**Fig. 2B and C**). Non-infectious spirochetes were never observed escaping the microvasculature. These observations indicated that early-stage interaction events were essential for sustained association and vascular escape. These observations also demonstrated that microvascular interactions were dependent on *B. burgdorferi* proteins expressed only in the infectious strain.

**Figure 3:**
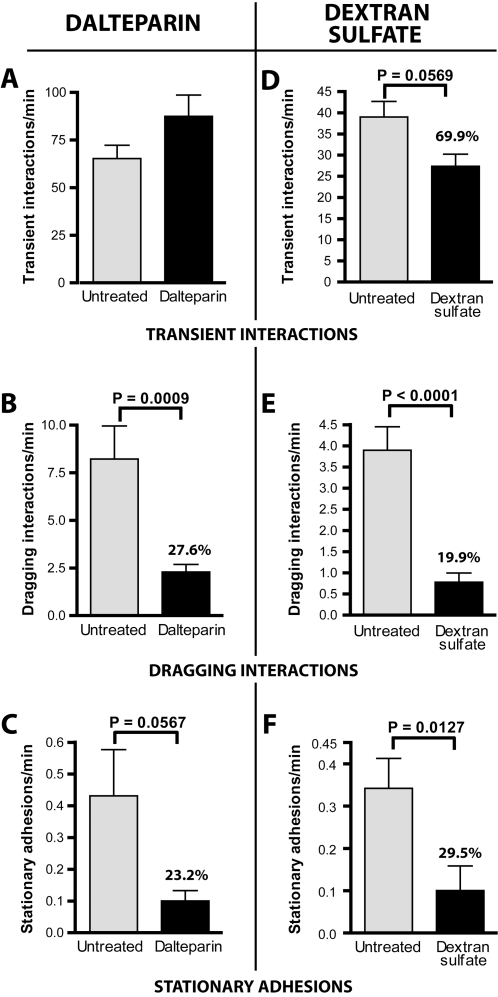
Role of host GAGs in spirochete microvascular interactions *in vivo*. The role of host GAGs was examined by conventional IVM performed using infectious *B. burgdorferi* preincubated with dextran sulfate (D–F) or with mice pre-injected with dalteparin, a therapeutic heparin compound (A–C). The percentages above the bars for dalteparin- or dextran sulfate-treated spirochetes indicate the treatment group interaction rate expressed as a percentage of the interaction rate of untreated spirochetes. For the dextran sulfate experiments, a total of 7,987 interactions in 96 venules from 14 mice (n = 8 and n = 6, respectively, for experiments performed with untreated and dextran sulfate-treated *B. burgdorferi*) were analyzed. Microvascular interactions were measured between 5 and 45 minutes after spirochete injection. Average time after injection: untreated (17.8−/+9.8 min), treated (18.1−/+9.5 min). For the dalteparin experiments, a total of 13,951 interactions in 77 venules from 13 mice (n = 7 and n = 6, respectively, for experiments performed with untreated and dalteparin-treated mice) were analyzed. Microvascular interactions were measured between 5 and 45 minutes after spirochete injection. Average time after injection: untreated (15.7−/+6.8 min), treated (16.3−/+7.1 min).

Many bacterially-encoded proteins interact with host cells via GAGs [29], and a number of previous *in vitro* studies performed under static conditions have found that *B. burgdorferi* can bind to GAGs and that exogenously applied GAGs can competitively inhibit interaction of *B. burgdorferi* with cell monolayers [13],[15],[30],[31]. Therefore, we investigated the potential role of endothelial host cell GAGs in spirochete microvascular association *in vivo*. Interaction rates were first examined in the presence and absence of a therapeutic low molecular weight heparin compound, dalteparin (Fragmin, average molecular weight 5 kDa). Dalteparin was used at a concentration previously shown to block leukocyte rolling *in vivo* [32]. Dalteparin (200 µl of a 25 I.U./µl solution) was injected via the femoral vein 15 minutes before intravenous inoculation with infectious spirochetes (see **Materials and Methods**). As shown in **Fig. 3A**, dalteparin treatment did not cause any significant change in transient interactions between fluorescent, infectious *B. burgdorferi* and the vascular endothelium. However, dragging interactions were significantly reduced by 72% (**Fig. 3B**) while the number of stationary adhesions were also reduced by dalteparin treatment to a similar extent (**Fig.** **3C**, 76% of controls).

Similar experiments were performed with dextran sulphate (**Fig. 3D–F**), a 500 kDa high molecular weight GAG analogue, which interacts *in vitro* with infectious but not non-infectious *B. burgdorferi* [30]. In these experiments, dextran sulfate was incubated with infectious *B. burgdorferi* for 30 minutes, followed by extensive washing, prior to *B. burgdorferi* administration to the animal, as this compound was toxic when injected directly into the mouse bloodstream. The concentration of dextran sulfate used in preincubations (20 µg/ml) was the dose that has been previously shown *in vitro* to maximally inhibit *B. burgdorferi* interaction with endothelial cells without altering spirochete morphology or motility [30],[31]. Preincubation of infectious *B. burgdorferi* with dextran sulfate caused a slight (30%) reduction in the number of transient interactions, but dragging interactions were reduced by 80% (**Fig. 3D and E**). A similar reduction in the number of stationary adhesions was also observed (**Fig. 3F**).

The results from the dalteparin and dextran sulphate experiments indicated that host GAGs play an important role in dragging interactions between *B. burgdorferi* and the microvascular endothelium *in vivo*, and that competition with a high molecular weight GAG analogue (dextran sulphate) also inhibited transient interactions. The similar levels of inhibition of dragging interactions and stationary adhesions caused by treatment with GAGs suggested that reductions in stationary adhesion were the result of inhibition of dragging. This in turn implied that additional host and spirochete molecules might contribute to stationary adhesion. However, the results of these experiments alone did not rule out the possibility that GAGs played a role in stationary adhesion.

### The *B. burgdorferi* GAG- and Fn-binding protein BBK32 is sufficient for transient and dragging microvascular interactions *in vivo*

**Figure 4:**
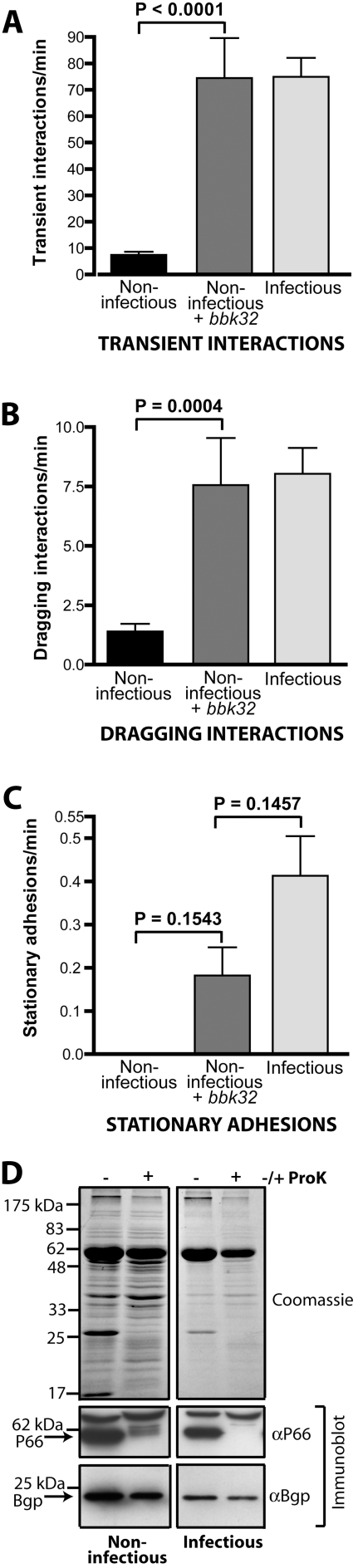
Role of *B. burgdorferi* GAG- and Fn-binding protein BBK32 in spirochete-microvascular interactions *in vivo*. (A–C) Interaction rates of three *B. burgdorferi* strains, infectious, non-infectious and the non-infectious strain with GFP and BBK32 expressed from the same plasmid (*bbk32* knock-in), as analyzed using conventional IVM. A total of 19,380 interactions in 81 venules from 16 mice (n = 9 infectious; n = 3 non-infectious; n = 4 non-infectious+*bbk32*) were analyzed. Microvascular interactions were measured between 5 and 45 minutes after spirochete injection. Average time after injection: infectious (13.4−/+9.9 min), non-infectious (12.1−/+4.8 min), *bbk32* knock-in (13.0−/+5.8 min). D) Expression and surface localization of adhesins P66 and Bgp in non-infectious and infectious strains, as determined by immunoblotting. The top panel shows total protein loaded for each strain, detected by Coomassie staining of the SDS-PAGE gel. To identify cell surface localized proteins, cell pellets were incubated in the presence (+) or absence (−) of proteinase K (ProK) before lysis. Proteinase K treatment resulted in a dramatic decrease in the level of P66 in both the infectious and non-infectious strains used here. Bgp showed a lesser, but similar reduction in both the infectious and non-infectious strain following treatment with Proteinase K.

Many bacterial adhesins can interact with GAGs, either directly, or indirectly via their association with host molecules such as fibronectin, regulators of the complement cascade and components of the coagulation system; furthermore, GAGs can act as bridging molecules that facilitate interactions between pathogen adhesins and host receptors [29]. In an effort to identify spirochete adhesins mediating GAG-dependent microvascular interactions *in vivo*, we PCR-amplified and sequenced all candidate *B. burgdorferi* adhesin genes identified to date [16], [17], [19], [21]–[25], using genomic DNA extracted from our fluorescent infectious and non-infectious strains (**Table S1**). This approach indicated that the genes encoding BBK32, VlsE, OspF, ErpL and ErpK were absent or mutated in the non-infectious strain (**Table S1**). It is possible that VlsE, OspF, ErpL and ErpK could mediate GAG-dependent host interactions directly or through recruitment of host molecules such as complement cascade regulators; however, interaction of these proteins with GAGs has not been directly demonstrated. In contrast, BBK32 has recently been shown to bind to host GAGs and to rescue the ability of non-infectious *B. burgdorferi* to interact with endothelial cells *in vitro* [25]. It was, therefore, of interest to investigate a possible role for BBK32 in *B. burgdorferi* interactions with the microvasculature in the living mouse. The *bbk32* coding sequence, under the control of the *ospC* promoter [25], was cloned into the GFP expression construct and the resulting plasmid was used to transform the parental non-infectious *B. burgdorferi* strain. Both parental and complemented strains had the same endogenous plasmid content (data not shown). Expression of BBK32 in the complemented strain was lower than the expression observed in the infectious strain (8.0−/+1.8%), but even this reduced expression was sufficient to restore transient and dragging interactions to the level observed with the infectious strain (**Fig. 4A and B**). However, stationary adhesion rates in the *bbk32* complementation strain did not reach the same levels as in the infectious strain (**Fig. 4C**), implying either a greater dependence upon BBK32 or a dependence upon additional spirochete factors that were missing in the complemented non-infectious strain. Attempts to genetically disrupt the *bbk32* locus in the infectious strain were successful, but did not result in usable constructs due to loss of endogenous plasmids (lp28-1 and others) in all recovered strains.

PCR amplification and sequencing of all candidate *B. burgdorferi* adhesin genes identified to date also indicated that other candidate adhesin genes (*bbf32, bbk2.10, bbO39 and bbm38*, encoding VlsE, OspF, ErpL and ErpK, respectively) were absent or mutated in the non-infectious parental strain (**Table S1**). Hence, these genes were not essential for transient and dragging interactions with the microvasculature of murine skin *in vivo*, since expression of BBK32 alone in this strain was sufficient to restore transient and dragging interactions. However, the possibility still exists that some of these genes play a role in stationary adhesion.

Examination of the sequence, expression and localization of two other major adhesins, P66 and Bgp, which have been shown to associate with integrins and GAGs respectively under static conditions *in vitro*, indicated that these proteins were expressed and localized similarly in non-infectious and infectious strains, and were not mutated (**Fig. 4D**; **Table S1**). Therefore, neither P66 nor Bgp expression nor localization was sufficient for transient or dragging interactions in the absence of BBK32 expression. Although the genomic sequence of the P66- and Bgp-encoding genes was identical *in* both infectious and non-infectious strains, it remains possible that secondary mutations elsewhere in the genome of non-infectious *B. burgdorferi* could have negatively affected transient and dragging interactions.

### Plasma Fn is essential for *B. burgdorferi* transient and dragging microvascular interactions *in vivo*

**Figure 5:**
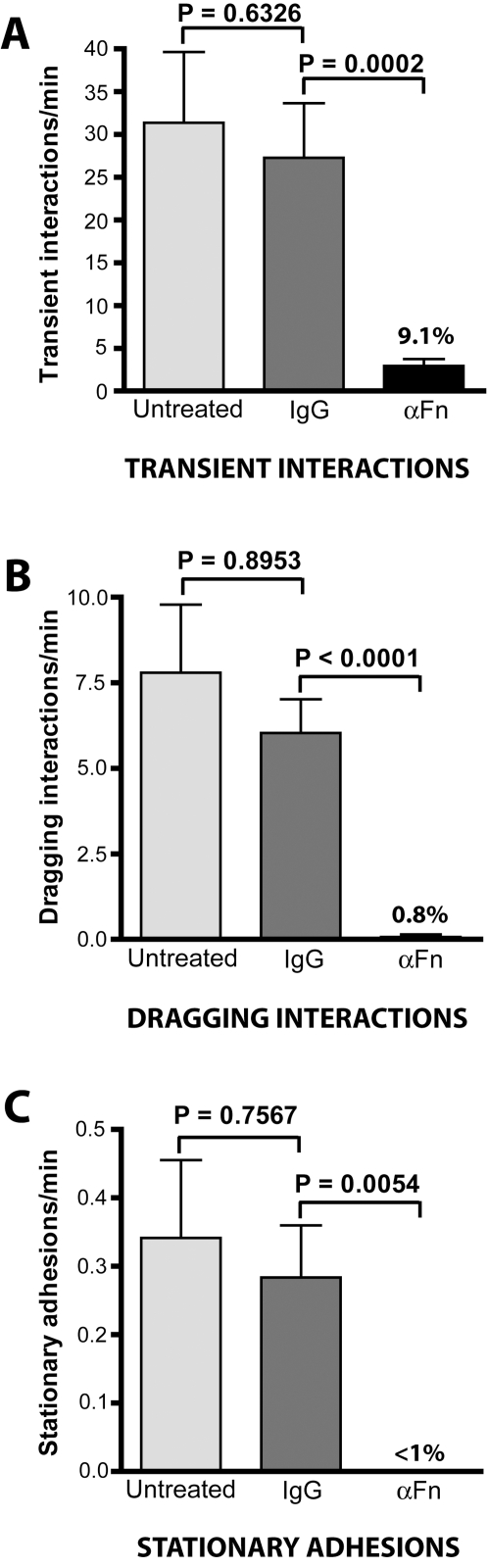
Role of host Fn in spirochete-microvascular interactions *in vivo*. (A–C) The role of host Fn in microvascular interactions was examined by conventional IVM performed using infectious *B. burgdorferi* preincubated with goat IgGs against rabbit Fn (αFn) or with non-specific goat IgGs. Non-specific or αFn IgGs were also injected directly into the mouse blood stream immediately before inoculation with spirochetes, and the effect of antibody on *B. burgdorferi* interactions was measured for up to 20 minutes after spirochete injection. The percentages above the bars for Fn antibody treatments indicate the interaction rate expressed as a percentage of the interaction rate of untreated spirochetes. A total of 2,614 interactions in 49 venules from 12 mice (n = 4 each for experimental group) were analyzed. Average time after injection: untreated (9.4−/+4.4 min), IgG (10.4−/+4.9 min), αFn (10.5−/+4.8 min).

Because BBK32 binds Fn in addition to GAGs, we also investigated a possible role for Fn in the adhesion of *B. burgdorferi* to the endothelium *in vivo*. Rabbit serum, which contains fibronectin, is an important component of the BSK-II medium used to propagate *B. burgdorferi*; therefore, we investigated whether antibodies to rabbit plasma Fn could disrupt *B. burgdorferi* microvascular interactions *in vivo*. Anti-Fn IgGs did not alter spirochete morphology or motility *in vitro*, implying that they were not toxic to *B. burgdorferi*. The tethering, dragging and stationary interactions/min for infectious *B. burgdorferi* treated with αFn IgGs were compared to the interaction rates of untreated spirochetes, and of spirochetes treated with nonspecific goat IgGs (**Fig. 5**). Preincubation of infectious spirochetes for 20 minutes with the IgG fraction of goat antiserum to rabbit plasma Fn, together with intravenous injection of this IgG fraction into the blood stream of mice, reduced transient and dragging microvascular interactions by 92% and 99%, respectively (**Fig. 5A and B**). When the same treatment regimen was performed using nonspecific goat IgGs, no effect on interaction rates was observed, indicating that the reduction in interactions following treatment with αFn IgGs was specific. Although stationary adhesions were essentially abolished by the αFn treatment (**Fig. 5C**), the reduction in transient and dragging interaction rates was so great that we could not determine if stationary adhesion rates were specifically affected by treatment with anti-Fn IgGs. Interestingly, interaction rates returned to normal levels 15–20 minutes after injection of the spirochetes and antibody (data not shown), suggesting that antibody-blocked rabbit Fn bound to spirochetes might have been replaced by mouse Fn *in vivo*, thus restoring microvascular interactions. The long population doubling time of *B. burgdorferi* (6–8h) precludes the possibility that restored interaction rates were caused by spirochete replication. The dramatic reduction in transient and dragging interactions resulting from Fn antibody treatment suggested that *B. burgdorferi* exploits host Fn for these interactions with the host microvasculature *in vivo*.

### Fibronectin sequences that bind heparin but not RGD-dependent integrins mediate transient and dragging microvascular interactions *in vivo*

Although experiments performed with anti-Fn IgGs suggested that Fn played a major role in the initiation of microvascular interactions, it was possible that IgG-dependent inhibition was partly a result of factors such as steric hindrance of interactions by bulky IgGs. Therefore, we also investigated the Fn dependence of interactions using Fn peptides. Fn is a structurally and functionally complex molecule (reviewed in [33]). Briefly, the N-terminal Type I Fn repeats and gelatin-binding region interact with Fn-binding proteins from *B. burgdorferi*, *Staphylococci* and *Streptococci in vitro* [16],[34],[35]. The central cell-binding domain contains multiple integrin-binding sites, including the canonical RGD sequence, which binds to most integrins that have been implicated in *B. burgdorferi*-host cell interactions to date [17],[19],[33]. Finally, the Fn C-terminus contains a high affinity heparin-binding domain that also interacts with host cell GAGs [33].

**Figure 6:**
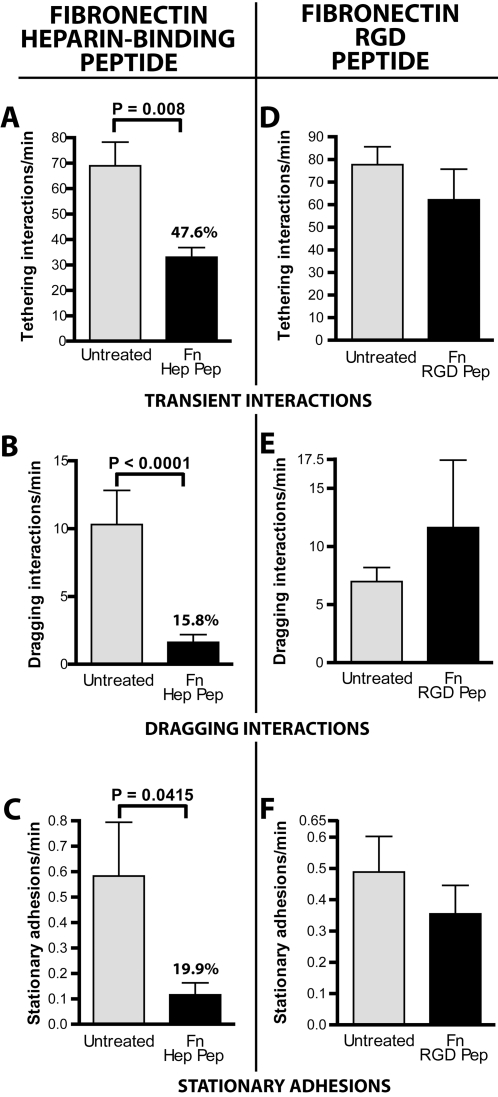
Contributions of GAG-binding sequences and integrin-binding sequences in Fn to spirochete-microvascular interactions *in vivo*. The roles of host glycosaminoglycans (GAGs) and integrins in Fn-mediated spirochete interactions with the microvasculature *in vivo* were examined by injecting mice with peptides corresponding, respectively, to a portion of the Fn heparin-binding domain (A–C) or the Fn RGD sequence (D–F). Microvascular interactions of infectious *B. burgdorferi* were examined by conventional IVM, for up to 20 minutes after intravenous inoculation of spirochetes. The percentages above the bars for peptide treatments indicate the interaction rate expressed as a percentage of the interaction rate of untreated spirochetes. For the heparin-binding Fn peptide experiments, a total of 8,346 interactions in 64 venules from 11 mice (n = 5 and n = 6, respectively, for experiments performed with untreated and peptide-treated mice) were analyzed. Average time after injection: untreated (10.8−/+3.6 min), treated (10.0−/+4.2 min). For the integrin-blocking Fn RGD peptide experiments, a total of 22,094 interactions in 89 venules from 16 mice (n = 9 and n = 7, respectively, for experiments performed with untreated and peptide-treated mice) were analyzed. Average time after injection: untreated (13.2−/+9.7 min), treated (12.4−/+6.0 min).

To investigate endothelial cell molecules associating with spirochete-bound Fn, we used peptides derived from the C-terminal heparin-binding domain and the integrin-interacting cell-binding domain in an attempt to block *B. burgdorferi-*microvascular interactions *in vivo* (**Fig. 6**). The heparin domain peptide (FN-C/H II: KNNQKSEPLIGRKKT) inhibits Fn-mediated cell adhesion and heparan sulfate binding [36], and the GRGDS cell-binding domain peptide is a well-studied competitive antagonist of integrin binding [37] that also inhibits *B. burgdorferi* interactions with integrins α_IIb_β_3_, α_v_β_3_ and α_5_β_1_ *in vitro* [15]. Peptides were injected via the femoral vein immediately before inoculation with infectious spirochetes, at concentrations (∼50 µg/ml of circulating blood) that disrupt leukocyte adhesion and recruitment *in vivo* [38]. Microvascular interactions in dermal postcapillary venules were recorded for no longer than 20 minutes after injection of peptide as the effect of peptide treatment on interaction rates was diminished at later time points, presumably because linear peptides are rapidly cleared from the mouse circulation [39].

Intravenous injection of 100 µg of the heparin-binding domain peptide reduced transient interaction rates by 52%, and impaired both dragging interactions and stationary adhesion levels by 84%, confirming the role of GAGs in early stages of microvascular interaction (**Fig. 6A–C**). In contrast, competition with the RGD peptide did not significantly inhibit any class of interaction (**Fig. 6D–F**), even though the estimated final concentration of this peptide in the mouse circulation (100 µM) was twice as high as the dose known to reduce *in vitro B. burgdorferi*-integrin interactions by at least 75% *in vitro* [15]. Administration of twice as much RGD peptide (∼200 µM final concentration) did not inhibit interactions, nor did intravenous administration of anti-CD41 and CD49e antibodies that respectively target RGD-dependent *B. burgdorferi*-interacting integrins containing α_IIb_ or α_5_ chains (platelet glycoprotein α_IIb_β_3_ and the α_5_β_1_ Fn receptor; data not shown). The effect of treatment with antibodies to α_v_β_3_ was not examined; however, since treatment with RGD peptide *in vitro* has been shown to strongly inhibit *B. burgdorferi* binding to this integrin as well as glycoprotein α_IIb_β_3_ and integrin α_5_β_1_, it seems unlikely that integrin α_v_β_3_ mediated the early stages of microvascular interactions. The conclusion that RGD-dependent integrins are not required for microvascular recruitment is consistent with the localization of known *B. burgdorferi*-associating RGD-dependent integrins, which are found at sites of endothelial attachment to extracellular matrix, and not in the lumen [15]. Collectively, these results implied that Fn-dependent transient and dragging interactions *in vivo* were mediated by host GAGs and not by RGD-dependent integrin interactions.

## Discussion

**Figure 7:**
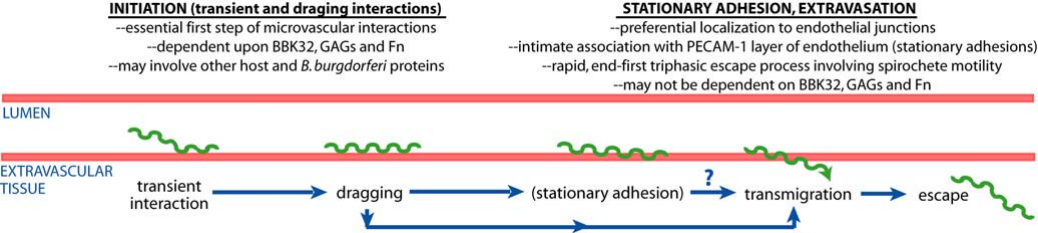
Schematic summarizing the stages of infectious *B. burgdorferi* interaction with and escape from the microvasculature. Based upon our previous work [9] and this study, we propose that transient and dragging interactions together constitute the essential first step (initiation) of microvascular interactions. We also propose that initiation interactions are mechanistically distinct from downstream interaction events (described in our recent paper [9]) for two reasons: 1) stationary adhesions and transmigrating spirochetes localize to different sites on the endothelium than transient and dragging interactions, and 2) stationary adhesion appears to require host and/or spirochete molecules in addition to or other than BBK32, GAGs and Fn. The results reported in this study do not indicate whether BBK32-mediated initiation events are entirely dependent on BBK32-GAG interactions bridged by Fn, or whether direct BBK32-GAG interactions also contribute to initiation.

In this study we employed intravital microscopy, a live cell imaging technique commonly used to analyze leukocyte recruitment and tumor dissemination *in situ* [40],[41], to investigate the molecular basis of *B. burgdorferi* dissemination *in vivo*. Our results demonstrate that IVM can provide critical insight into the mechanisms of pathogen dissemination. **Fig. 7** provides a summary of the features of *B. burgdorferi* dissemination which we have identified using IVM, based on data described in this study and in a recent companion report [9].

This study revealed pivotal roles for the *B. burgdorferi* adhesin BBK32 as well as host GAGs and fibronectin in the initiation of spirochete-microvascular interactions (see below) (**Fig. 7**). Although other host and *B. burgdorferi* molecules may also contribute to microvascular interactions *in vivo*, the involvement of GAGs and Fn in this process is especially interesting, since a broad array of pathogens are known to interact *in vitro* with these host molecules in direct binding assays and tissue culture models (reviewed in [29], [42]–[45]. However, the potential contribution of these interactions to processes such as hematogenous dissemination has not been directly examined in living hosts.

### Evidence for mechanistically distinct stages in *B. burgdorferi* microvascular dissemination *in vivo*

We found that BBK32 and its host ligands Fn and GAGs played major roles in transient and dragging interactions. Although we cannot rule out the possibility that these molecules also contribute to stationary adhesion, the results of the *bbk32* complementation experiments indicate that additional spirochete molecules are likely required for stationary adhesion. This implies that stationary adhesion is a mechanistically distinct step in *B. burgdorferi* dissemination. This conclusion is supported by our previous observations that: 1) stationary adhesions form primarily at endothelial junctions, whereas short-term interactions occur chiefly on endothelial cells themselves; and 2) stationary adhesions associate more intimately with the endothelium than short-term interactions and appear to traverse the surface of the endothelium when these cells are labeled with PECAM-1 (**Fig. 7**) [9]. These observations imply that *B. burgdorferi* dissemination shares functional similarities with the sequence of events that constitute the leukocyte recruitment cascade [46],[47], as well as the events associated with dissemination of circulating tumor cells [48]. Leukocyte recruitment is initiated by selectin-mediated tethering and rolling interactions that permit firm adhesion, which is mediated by integrins. The initiation phase of leukocyte recruitment is a rate-limiting step, as it is essential for all subsequent events in the recruitment cascade. Similarly, we propose that transient and dragging interactions mediated by GAGs and Fn together constitute the corresponding initiation phase of *B. burgdorferi* dissemination, while other host and spirochete molecules become essential at the stationary adhesion phase.

Though our data indicated that transient and dragging associations were mediated by the same host and spirochete molecules, the observation that the low molecular weight heparin dalteparin inhibited only dragging interactions was surprising. The reason for this is currently unknown, but may result from differences in total charge, chain length and chemical composition of the carbohydrate moieties.

### A role for Fn, GAGs and BBK32 in the initiation of *B. burgdorferi* microvascular interactions

This study identified a central role for the *B. burgdorferi* protein BBK32, host GAGs and Fn in the initiation of microvascular interactions. This observation was unexpected, since previous studies have shown that genetic disruption of *bbk32* attenuates but does not abolish infectivity [49],[50]. However, *bbk32* disruption mutants still bind Fn [49],[50], implying that other functionally redundant Fn-binding proteins in *B. burgdorferi* might also mediate the initiation of dissemination. The simplest interpretation of our data is that initiation is mediated by BBK32 interactions with GAGs, either independently or via a fibronectin bridge. It is possible that initiation might also be mediated by RGD-independent integrins such as α_3_β_1_, which interacts with Fn, GAGs and the *B. burgdorferi* protein BBB07 [18],[19]; however, this integrin is expressed at endothelial junctions [51] implying that it is more likely to mediate stationary adhesion or extravasation than initiation interactions. Furthermore, the activation of adhesive properties by endothelial integrins generally requires endothelial activation [46], which is not detected in the short time frame of our experiments [9]. Taken together, these data make it unlikely that integrins play a role in the initiation of vascular adhesion.

All molecules to date implicated in tethering under shear force conditions (selectins, von Willebrand factor the *E. coli* FimH adhesin) interact with sugar-containing ligands [52],[53], suggesting that Fn-dependent or -independent interactions between BBK32 and GAGs might promote *B. burgdorferi* tethering by a similar mechanism. The affinity of BBK32 for GAGs is unknown, but in the absence of shear forces BBK32 associates with high specificity and probable high affinity to the Fn N-terminus via a tandem β-zipper mechanism shared with Fn-binding proteins of *Staphylococcus aureus* and *Streptococcus pyogenes* [34],[35],[54],[55]. In the absence of shear forces, the affinity of plasma Fn for heparin (K_d_ = 0.1–1.0 µM) is within the affinity range of P- and E-selectins for their ligands (K_d_ = 1.5 µM and 109 µm, respectively) [56],[57], suggesting that BBK32-, GAG- and Fn-dependent initiation interactions may be mechanistically feasible.

Although under shear stress conditions Fn does not bind to the leukocyte Fn receptor VLA-4, which mediates tethering to endothelial VCAM-1 under flow [58], previous reports indicate that both platelets and *Mycobacterium tuberculosis* can bind to immobilized Fn *in vitro* under shear stress conditions that mimic those found in postcapillary venules [59],[60]; interestingly, platelet-Fn interactions are almost completely blocked by treatment with unfractionated or high molecular weight heparin [60]. This suggests the possibility that Fn-dependent tethering interactions entail cooperative GAG binding, a conclusion that is consistent with our observation that expression of the Fn- and GAG-binding BBK32 protein was sufficient to restore initiation interactions to wild-type levels.

Another possibility is that BBK32-induced conformational changes in Fn might facilitate Fn- and GAG-dependent tethering interactions. This hypothesis stems from recent data from the Höök laboratory indicating that BBK32 binding to Fn induces the formation of superfibronectin (S. Prabhakaran and M. Höök, personal communication), a high molecular weight Fn complex that substantially enhances adhesion of cells to Fn by integrin-dependent and independent mechanisms [61]. Further analysis of the precise mechanisms underlying BBK32-, Fn- and GAG-dependent dissemination under shear force conditions will be required.

The results of this study emphasize the importance of directly investigating host-pathogen interactions in a native context where major regulators of interaction such as fluid shear stress are present. The methodology and observations presented here provide the first direct insight into the role of host GAGs, Fn and a *B. burgdorferi* protein that binds both of these host components, in host microvascular interactions *in situ*. These results may have broad-reaching implications for our understanding of processes underlying the dissemination of a variety of other bacterial pathogens that interact with Fn and GAGs.

## Materials and Methods

### Construction of the BBK32 expression plasmid pTM170

Plasmid pTM170 was constructed by PCR amplification of the P*ospC*-driven *bbk32* cassette from pBBK32 [25] with flanking *Kpn*I and *Fsp*I sites, using primers B1093 (5′-GGTACCTTAATTTTAGCATATTTGGCTTTG-3′) and B1094 (5′-GGCCTGCGCATTAGTACCAAACGCCATTCTTG-3′). The PCR product was cloned into the GeneJet plasmid, using the Gene Jet blunt cloning kit (MBI Fermentas) to generate pTM169. The *Kpn*I/*Fsp*I-digested pTM169 insert was cloned into the KpnI/FspI sites of the GFP-encoding plasmid pTM61 [9] to yield pTM170.

### *B. burgdorferi* transformations and screening

*B. burgdorferi* strains used in this study were GCB705 (non-infectious strain B31-A transformed with pTM61) [9],[62], GCB726 (infectious *B. burgdorferi* strain B31 5A4 NP1 transformed with pTM61) [9],[63] and GCB769 (non-infectious B31-A transformed with pTM170). The plasmid content of these strains is noted in **Table S1**. All strains were grown in BSK-II medium prepared in-house [64]. Electrocompetent *B. burgdorferi* strains were prepared as described [9],[65]. Liquid plating transformations were performed with 50 µg pTM61 or pTM170 in the presence of 100 µg/ml gentamycin as described [66],[67]. Gentamycin-resistant *B. burgdorferi* clones were screened for: 1) the presence of *aacC1* sequences by colony screening PCR performed with primers B348 and B349 as described [68]; 2) GFP expression by conventional epifluorescence microscopy, and 3) BBK32 expression, as detected by immunoblotting (for *bbk32* complementation strains). The presence of plasmids in non-integrated form in fluorescent strains was confirmed by agarose gel electrophoresis of total genomic DNA prepared on a small scale as described [69]. PCR screening for native plasmid content was performed as described [68],[70].

### PCR amplification and sequencing of candidate genes

Gene sequences were amplified from genomic DNA preparations of GCB705 and GCB726. PCR was performed with Phusion DNA polymerase (NEB, Pickering, Ontario, Canada), according to manufacturer's instructions. Sequencing was performed by the University of Calgary DNA Services. All primers used for PCR amplification and sequencing are provided in **Table S1**.

### Protein expression and localization studies

The expression and outer membrane localization of adhesins P66 and Bgp were analyzed as previously described [16],[25]. Briefly, for each strain, two pellets containing 5×10^7^ spirochetes were washed twice with PBS+2% BSA. PBS containing 5 mM MgCl_2_ was added without dislodging pellets. Proteinase K was added to one pellet to a concentration of 4 mg/ml. After 30 min incubation at room temperature, reactions were stopped with 150 µg phenylmethylsulfonyl fluoride, spirochetes were pelleted and washed twice with PBS+0.2% BSA, and pellets were lysed using SDS-PAGE loading dye. Proteins were resolved by electrophoresis on 12% SDS-PAGE gels, transferred to nitrocellulose membranes, followed by immunoblotting with antibodies to P66, Bgp or BBK32, as previously described [17],[22].

### Preparation of fluorescent *B. burgdorferi* for direct bloodstream injection, surgical preparations and intravital microscopy conditions

These conditions have been described in detail previously [9]. Quantification of spirochete interactions was performed as recently described [9]. All animal studies were carried out in accordance with the guidelines of the University of Calgary Animal Research Centre.

### Quantification of leukocyte recruitment in dermal microvasculature of mice in the presence and absence of infectious *B. burgdorferi*

Leukocyte recruitment studies were carried out as previously described [28]. Briefly, animals were injected with 50 µl of 0.05% (i.v.) rhodamine 6G (Sigma-Aldrich). Fluorescence was visualized by epi-illumination using 510 and 560 filters. Leukocytes were considered adherent to the venular endothelium if they remained stationary for 30 s or longer. Experiments were performed in mice that had been intravenously inoculated with 4×10^8^ infectious *B. burgdorferi* grown for 48h in 1% mouse blood, as previously described, and also with mice that were not inoculated with spirochetes. Leukocyte adhesions were counted in the dermal postcapillary venules of infected and non-infected mice from 5 minutes after injection of spirochetes and/or rhodamine until at least 1 hour from injection, in order to monitor leukocyte recruitment during the time frame that is used for all experiments reported in this study.

### Fibronectin antibody experiments

GCB726 spirochetes prepared as described above were resuspended to 2×10^9^/ml in PBS. The IgG fraction of polyclonal goat anti-rabbit plasma Fn serum or non-specific goat IgGs (Cappel/MP Biomedicals, Solon, OH) were added to 1 mg/ml final. After mixing for 30 min at room temperature, spirochetes were directly injected into the mouse bloodstream, along with 2 mg of Fn antiserum IgGs or non-specific IgGs.

### Fn peptide experiments

GCB726 spirochetes were prepared and injected as described above, together with 100 µg of GRGDS or FN-C/H II peptide (Sigma Canada, Oakville, ON; catalogue numbers G4391 and F7049, respectively), injected via the femoral vein. Peptides injected at this amount are known to disrupt leukocyte adhesion and recruitment *in vivo* [38].

### Dalteparin and dextran sulfate experiments

Two hundred µl of a 25 I.U./µl solution of dalteparin (Fragmin: Pfizer Canada, Kirkland, PQ) were injected via the femoral vein 15 minutes before intravenous inoculation with infectious spirochetes. This concentration has previously been shown to inhibit leukocyte rolling *in vivo* [32]. Dextran sulfate-treated spirochetes were incubated with 20 µg/ml dextran sulfate (500 kDa; Fisher Scientific Canada, Ottawa, ON) in a final volume of 100 ml PBS for 30 min at RT°C, followed by 2 100ml washes with PBS. Spirochetes were resuspended to 2×10^9^/ml in PBS, and injected as previously described [9]. The concentration of dextran sulfate used in these preincubations (20 µg/ml) is the dose that maximally inhibits *B. burgdorferi* interaction with endothelial cells *in vitro*, and does not affect spirochete morphology or motility [30],[31].

### Integrin-targeting antibody experiments

One hundred µg anti-CD41 monoclonal Ab (Clone MwReg30; Becton Dickinson, San Diego, CA), or 20 µg CD49e monoclonal Ab (clone 5H10-27; Pharmingen, Oxford, UK) were intravenously administered immediately prior to injection of spirochetes. These quantities of anti-CD41 and CD49e antibodies are those that respectively protect against *Plasmodium berghei* infection *in vivo* [71], and which inhibit neutrophil migration *in vivo* [72].

### Statistics

For quantitative analysis, average and standard error values for different variables were calculated and plotted graphically for all vessels from all mice using GraphPad Prism 4.03 (GraphPad Software, Inc., San Diego, CA). Statistical significance was calculated in GraphPad Prism using a two-tailed non-parametric Mann Whitney t-test with a 95% confidence interval.

## Supporting Information

Table S1 PCR amplification and sequencing of candidate *B. burgdorferi* adhesin genes.
(0.05 MB DOC)

Video S1 Spinning disk confocal IVM video footage of fluorescent *B. burgdorferi* interacting with a postcapillary venule of the skin vasculature. Elapsed time is shown at the top right and the scale at bottom left. Direction of blood flow is down and to the left.
(1.1 MB SWF)

Video S2 Conventional epifluorescence IVM video footage of fluorescent *B. burgdorferi* interacting with a postcapillary venule of the skin vasculature. The video is shown in real time (time indicated at the bottom). Blood flow direction is to the right and up.
(1.9 MB SWF)
