# Supplementary material for: Molecular Mechanisms Involved in Vascular Interactions of the Lyme Disease Pathogen in a Living Host
Source: PLoS Pathog. 2008 Oct 3;4(10):e1000169. doi: 10.1371/journal.ppat.1000169 (PMC2542414; doi:10.1371/journal.ppat.1000169)
Supplement: Table S1 — PCR amplification and sequencing of candidate B. burgdorferi adhesin genes. (0.05 MB DOC) [file ppat.1000169.s001.doc]

| **Supplementary Table 1: PCR amplification and sequencing of candidate *B. burgdorferi* adhesin genes** | | | | | | | |
| --- | --- | --- | --- | --- | --- | --- | --- |
| **Candidate adhesin** | **Site of gene** | **Plasmid present?** | | **Gene present by PCR?** | | **Mutation in GCB705?** | **PCR amplification and sequencing primers** |
|  |  | ***GCB 726*** | ***GCB 705*** | ***GCB 726*** | ***GCB 705*** |  |  |
| *bbf32* | lp 28-1 | + | no | n/a | n/a | n/a | n/a |
| *bbk32* | lp 36 | + | no | n/a | n/a | n/a | n/a |
| *bbk2.10* | lp 36 | + | no | n/a | n/a | n/a | n/a |
| *bba24* | lp 54 | + | + | + | + | no | B1044 5’-CCGGATCCAAAATCACAAGCCAGATTGCAT-3’  B1045 5’-CCTGCGCATATGTCTTGATTATCGGGCGA-3’ |
| *bba25* | lp 54 | + | + | + | + | no |
| *bba52* | lp 54 | + | + | + | + | no | B1055 5’-TTAAAGGCCTTGGGTACATTC-3’  B1056 5’-TTATTCTGGCTTTAAAGCCTAAA-3’ |
| *bba66* | lp 54 | + | + | + | + | 2 coding mutations | B1057 5’-TTTGAAATAGAACAATAAAAAAAAGA-3’  B1058 5’-CACTTGATATTTTAAAAAGAGAAG-3’ |
| *bbb07* | cp 26 | + | + | + | + | no | B1095 5’-GAATTAAGCATTTTTGAAGAATTAA-3’  B1096 5’-TAAAAATCTTGATTTAAGAGAGTC-3’ |
| *bbb19* | cp 26 | + | + | + | + | no | B1059 5’-CAATTATTTGCATTCCATAACTA-3’  B1060 5’-AATAAAAAAATAAAAGCCAAGAAATC-3’ |
| *bbo39* | cp32-2/7 | + | + | + | no | n/a | B1268 5’-TTGGGTGTTCTCATTGCAT-3’  B1269 5’-TGGTCTTCTTGTTGACCAC-3’  B1270 5’-TTGTGCTGTTTTTGCATTGATG-3’  B1271 5’-TAATTCATCAGTATTGCTGTTGT-3’ |
| *bbs41* | cp 32-3 | + | + | + | + | n/a | B1061 5’-TTCTTGAAATAGTTTGAGCGTAA-3’  B1062 5’-GCTTTATTGCCTAAATTTCAGC-3’ |
| *bbm38* | cp 32-6 | + | no |  | n/a | n/a | n/a |
| *bb0108* | chromosome |  |  | + | + | no | B1097 5’-ATTAAGCATTGAAAAGGCCTTG-3’  B1098 5’-TGCGCTTTGATTAATATCAATGC-3’ |
| *bb0210* | chromosome |  |  | + | + | Premature stop codon | B1049 5’-CGATGGAATTGCAGTAGGGG-3’  B1050 5’-GTAGTTCCCTTAATATTGAACAT-3’  B1051 5’-GCACAATAACAGAAAACTACTC-3’  B1052 5’-TCGTTAGGTTTTTTTGCATTGG-3’  B1099 5’-GGGGGCTTTGGTTACTATAT-3’  B1100 5’-GCAACAAATTTCAAAAAGTAAATG-3’  B1101 5’-AGGCCTGGGCGTCATTGG-3’  B1102 5’-GCAATACAAATTGATAAAAATTATG -3’ |
| *bb0347* | chromosome |  |  | + | + | no | B1156 5’-GCTGATTAAAAGAAGTCCCAAGAG-3’  B1171 5’-TTTCACAAATTAATCTGTCAGGCA-3’  B1179 5’-ACATAAGCTCCAGGATAATCTC-3’ |
| *bb0385* | chromosome |  |  | + | + | no | B1266 5’-CCCTAAGTTTTACAACACCATCA-3’  B1267 5’-GACAAGATGGCTGTTTTTTATAAAGC-3’ |
| *bb0588* | chromosome |  |  | + | + | no | B1036 5’-ATTGTAATATGGATAGGATATTTTT-3’  B1037 5’-AAATATTCTTGCCTTCTTAAAAAC -3’ |
| *bb0603* | chromosome |  |  | + | + | no | B1033 5’-AAAAGATGCAAATGATAAATTTATAA-3’  B1034 5’-CATCTTTTGGAGCTAAATATAAG-3’  B1035 5’-ATTATATACATGTTATATATATGTTAA-3’ |
